# Psychotherapies in opioid use disorder: toward a step-care model

**DOI:** 10.1007/s00702-023-02720-8

**Published:** 2023-11-21

**Authors:** Amaury Durpoix, Julie Rolling, Romain Coutelle, Laurence Lalanne

**Affiliations:** 1 Addictology Department, Strasbourg University Hospital, 1, place de l’Hôpital, 67091 Strasbourg, France; 2 Psychiatry, Mental Health and Addictology Department, Strasbourg University Hospital, Strasbourg, France; 3 Regional Center for Psychotrauma Great East, Strasbourg, France; 4 INSERM U1114, Cognitive Neuropsychology, and Pathophysiology of Schizophrenia, Strasbourg, France; 5 Centre National de la Recherche Scientifique Unité Propre de Recherche 3212 (CNRS UPR 3212), Institute for Cellular and Integrative Neurosciences (INCI), Strasbourg, France; 6 Strasbourg University, Faculty of Medicine, Strasbourg, France; 7 Fédération de Médecine translationnelle de Strasbourg, Strasbourg, France

**Keywords:** Opioid use disorder, Opioid addiction, Psychotherapies, Psychosocial, Stepped-care model, Adolescents, Psychiatric comorbidities

## Abstract

Opioid use disorder (OUD) is characterized by a lack of control in opioid use, resulting in psychological distress and deficits in interpersonal and social functioning. OUD is often associated with psychiatric comorbidities that increase the severity of the disorder. The consequences of OUD are dramatic in terms of increased morbi-mortality. Specific medications and psychotherapies are essential tools not only in the treatment of OUD but also in the prevention of suicide and overdoses. In our review, we assess the different types of psychotherapies (counseling, motivational interviewing, contingency management, cognitive-behavioral therapy, and dialectical-behavior therapy) that are delivered to opioid users, either associated or un-associated with OUD medications and/or medications for psychiatric disabilities. We describe the application of these therapies first to adult opioid users and then to adolescents. This work led us to propose a stepped-care model of psychotherapies for OUD which provided information to assist clinicians in decision-making regarding the selection of psychotherapeutic strategies according to patients’ OUD severity.

## Introduction

In the past 15 years, opioid overdoses have been continuously rising; they currently represent a major public health crisis that largely affects North America (Hedegaard et al. 2015) and are now estimated at 5.4/100,000 in the USA. Moreover, one in 10 adolescents and young adults (15–24 years of age) who died in 2016 died of opioid-related causes (Gomes et al. 2018). In European countries, some indicators suggest the emergence of similar trends, with over 9000 fatal overdoses in 2016 (European Monitoring Centre for Drugs and Drug Addiction 2018). This public health problem highlights the need to develop innovative therapeutic strategies, both pharmaco-therapeutic and psychotherapeutic. Today, only two opioid medications of substitution are used in medical practice, namely methadone and buprenorphine, and they are always prescribed with a psychosocial follow-up. Moreover, peculiar psychotherapies, such as motivational interviewing, have been developed in addiction medicine with the goal of changing addictive behavior (Miller and Rollnick 1991). Psychotherapeutic support is therefore also an issue in treatment.

Opioid use disorder (OUD) is characterized by problematic opioid use (Hasin et al. 2013), the severity of which is defined by a lack of control in opioid use, resulting in psychological distress and deficits in interpersonal and social functioning. The consequences of OUD are dramatic in terms of increased morbi-mortality, with one’s life expectancy decreased by more than 10 years (Degenhardt et al. 2020).

Clinical observations further indicate that higher opioid consumption correlates with poorer outcomes. Studies have shown that most high-dose opioid users suffer from psychiatric disorders (dual diagnosis) with a heightened risk of overdose (Ranapurwala et al. 2018). Among psychiatric comorbidities, a recent meta-analysis revealed a prevalence of anxiety at approximately 29% (95% CI 24.0–33.3%), depression at 36.1% (95% CI 32.4–39.7%), post-traumatic stress disorders at 18.1% (95% CI 15.4–20.9%), ADHD at 20.9% (95% CI 15.7–26.2%), anti-social personality disorder at 33.6% (95% CI 29.1–38.0%), and borderline personality disorder at 18.2% (95% CI 13.4–23.1%) (Santo et al. 2022). These psychiatric comorbidities confer a vulnerability and increase the severity of OUD. For example, in depressed, compared with non-depressed, individuals, opioid therapy is misused for longer durations, with the objective of alleviating dysphoria stress and insomnia symptoms related to potential opioid antidepressant effects (Lutz and Kieffer 2013). Thus, regardless of the acute efficacy of opioids for psychiatric symptoms, their long-term repeated use precipitates vulnerable individuals into compulsive patterns of drug use and the development of OUD, thereby reinforcing concurrent psychiatric disorders and leading to de-socialization, precariousness, and dramatic personal and familial situations.

The psychiatric factors associated with OUD are not limited to adults but occur in adolescents. Among adolescents, various psychosocial risk factors have recently been associated with prescription opioid misuse, particularly among adolescents with a history of depressive episodes, compared with those without (1.5-fold higher risk of prescription opioid misuse) (Edlund et al. 2015). Boyd also found that adolescents who abused prescription opioids for drug use had more symptoms of affective disorders as well as more symptoms of anxiety, somatic disorders, attention disorders, and conduct disorders than adolescents who did not use opioids (Boyd et al. 2014). In addition, a history of childhood emotional or physical abuse (maltreatment) has been associated with recent prescription opioid misuse in early adulthood (Stein et al. 2017). This focus on adolescents is of special importance given that this population is one of the most vulnerable to opioid misuse (Windisch and Kreek 2020). Indeed, adolescence is the period of life when young people discover various addictive substances, while their brains are still developing. Differences in maturation between the prefrontal cortex and the limbic system are at the root of difficulties with emotional regulation, sensation-seeking, and the need for novelty and experimentation (Casey et al. 2008). These characteristics explain why adolescence is the period when drug use begins. Adolescents use drugs to alleviate distress, suffering, and discomfort associated with identity formation. They seek to ‘escape for a few moments/hours’. In addition, social phenomena reinforce the risk of drug use (e.g., imitation of other young people or the desire to fit in with a social group and respect its codes).

The frequent co-occurrence of psychiatric comorbidities or psychosocial risk factors and OUD raises the question of their treatment in adults as in adolescents. Regardless of specific medications for OUD and for their psychiatric comorbidities, psychotherapies are an essential tool for the treatment and prevention of suicide and overdoses. Numerous psychotherapies exist, some of which are particularly well suited to OUD. According to the APA (APA Dictionary of Psychology), psychotherapy refers to any psychological service provided by a trained professional that primarily uses forms of communication and interaction to assess, diagnose, and treat dysfunctional emotional reactions, ways of thinking, and behavior patterns (VandenBos 2015). Psychotherapy may be provided to individuals, couples, families, or members of a group. There are many types of psychotherapies, but generally they fall into four major categories: psychodynamic psychotherapy, cognitive therapy or behavior therapy, humanistic therapy, and integrative psychotherapy.

In our review, we assess different types of psychotherapies that are delivered to opioid users, either associated or un-associated with OUD medications and/or medications for psychiatric disabilities. We describe their application first to adult opioid users and then to adolescents. This literature review will allow us to determine what the most adapted type of psychotherapy is as a function of the complexity of OUD, its association with psychiatric comorbidities, and the risk of suicide and overdose. This, in turn, will enable us to examine the possibility of a stepped-care model of psychotherapies for OUD that provides information to assist clinicians in decision-making regarding selecting psychotherapeutic strategies according to patients’ OUD severity.

## Methods

This present narrative review is based on a thorough literature search through peer-reviewed journals. We searched the literature databases PubMed and Google Scholar on April 24, 2023, for peer-reviewed articles between January 1990 and April 2023 on psychotherapeutic and psychosocial interventions in OUD related to adolescents and adults. We used the search terms ‘psychosocial interventions’ OR ‘psychotherapies’ AND ‘opioid use disorder’ OR ‘substance use disorder’. Furthermore, we retrieved additional references related to (1) subtopics, (2) sources cited in the initially retrieved references and estimated as important, and (3) our own knowledge of the literature.

With this knowledge, we organized the psychotherapies and psychosocial interventions according to the complexity of the following clinical questions that address current issues in OUD presented in the introduction:

1. What is the minimum level of effective psychosocial interventions?
2. How should we manage in case of unmotivated patient for cares?
3. How should we manage in case of severe (poly)addictions?
4. How should we manage in case of a dual diagnosis?
5. How should we do in case of recurring self-harm behavior?

For each cited intervention, we summarize its principle, its efficacy in adult and adolescent populations, its prize, its training, and its limits.

## Results

### What is the minimum level of effective psychosocial interventions?

**Fig. 1:**
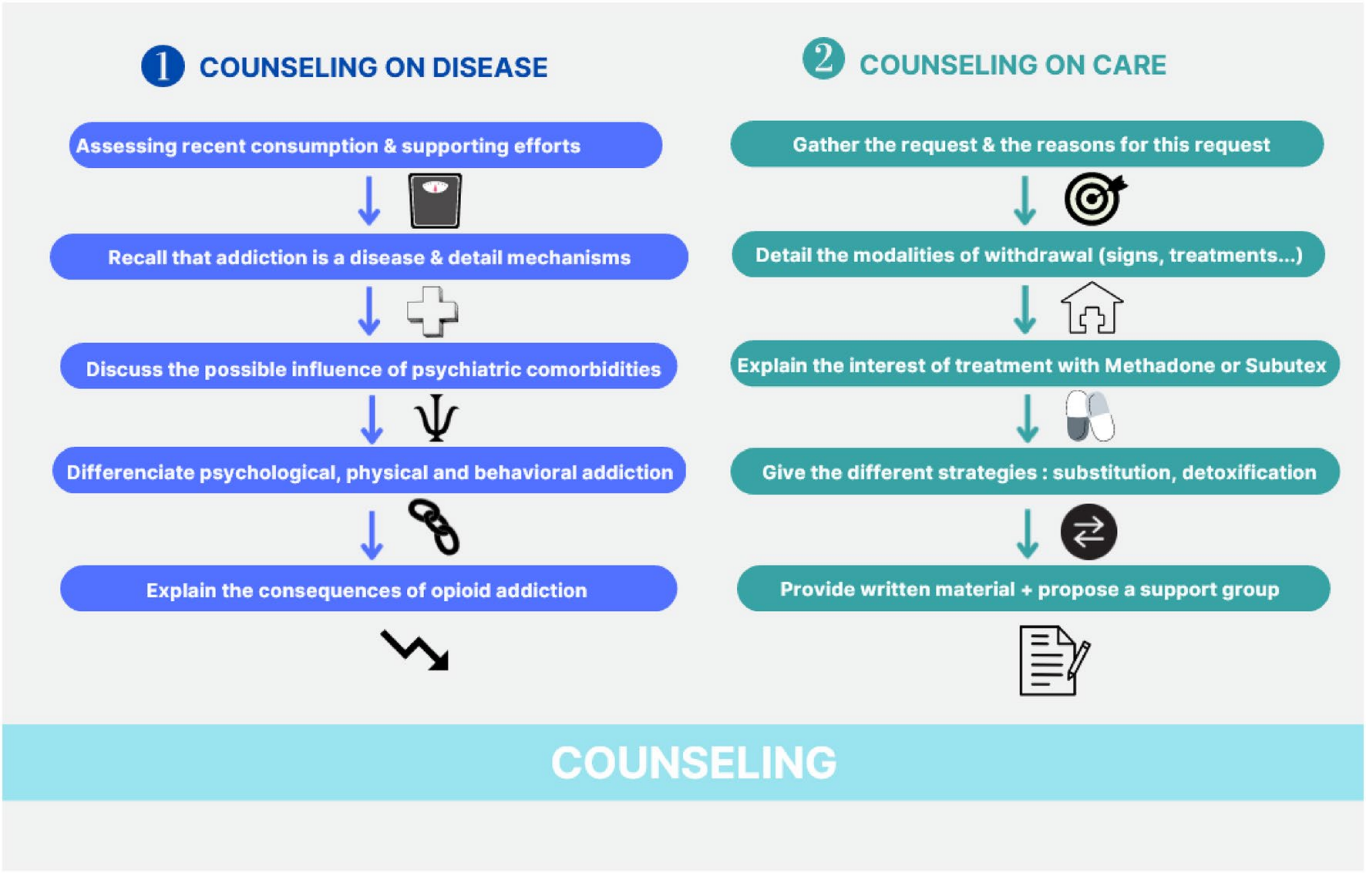
Principles of counseling: counseling goal is to improve knowledge about patient. Text corresponds to several different steps. Logos help to memorize

Psychosocial interventions include all interventions in which counseling or behavior management is used. In OUD, such interventions are always conducted and evaluated with opioid agonist therapy (OAT). An opioid agonist replaces the misused opioid substance by interfering with endogenous signaling within the opioid system (Lutz and Kieffer 2013; Noble et al. 2015). Buprenorphine and methadone are the pharmacological treatments prescribed to patients suffering from OUD. In studies, medical sessions accompanying the prescription of OAT have different names: medical management, individual counseling, and treatment as usual. The guidelines predominantly recommend counseling (Watan Pal et al. 2021) (see Fig. 1). To determine the most effective follow-up intensity, Fiellin et al. (2006) detail and standardize them. In their study, they recommend that sessions should cover recent drug use, efforts realized, support for these efforts, attendance in self-help groups, advice to reach or pursue abstinence, and results of weekly urinalysis. With the physician, the sessions should additionally cover an assessment of employment, legal, family, social, medical, and psychiatric problems related to addiction. In their study, patients had weekly medication dispensing, weekly 20-min meetings with trained primary care nurses, and monthly 20-min check-ups with a physician. When Fiellin et al. (2006) compared the group of patients who benefit from this intervention with other groups who benefit from a more intense intervention, they found no superiority of the latter group compared with the former. This intervention might be associated with mutual-help groups such as Narcotics Anonymous (Moos and Timko 2008).

For methadone maintenance, a recent meta-analysis even confirmed that counseling was one of the most effective psychosocial interventions (Wen et al. 2023). Controlled studies have demonstrated that OAT accompanied by a psychosocial intervention is highly effective. Without this follow-up intervention, the mortality rate is high: 13.8% at 10 years, 27.7% at 20 years, and 48.9% at 30 years (Hser et al. 2001; Shulman et al. 2019).With OAT, the mortality rate decreases by 75% compared with drug-free behavioral treatment and by more than twice compared with no treatment (Clark et al. 2011; Patel et al. 2021). These improvements in treatment retention decrease opioid use and increase patient survival (Gibson et al. 2008; Patel et al. 2021). Considering the efficacy of this treatment, which includes OAT with counseling in medical and psychosocial support, it is not expensive: approximately $6500 per year (NIDA 2021; Patel et al. 2021).

Given the physical and psychological upheavals induced by stopping a substance or switching to an OAT, some patients may refuse this first stage of care. Refusal can be active or, more often, passive, with the latter displaying as ambivalence. Ambivalence is a key symptom of addictive disorders that prevents opioid users from changing their habits and stopping drug use. Having valid information and offering effective treatment are not always sufficient. In this case, a second step is required.

### How should we manage in case of unmotivated patient for cares?

**Fig. 2:**
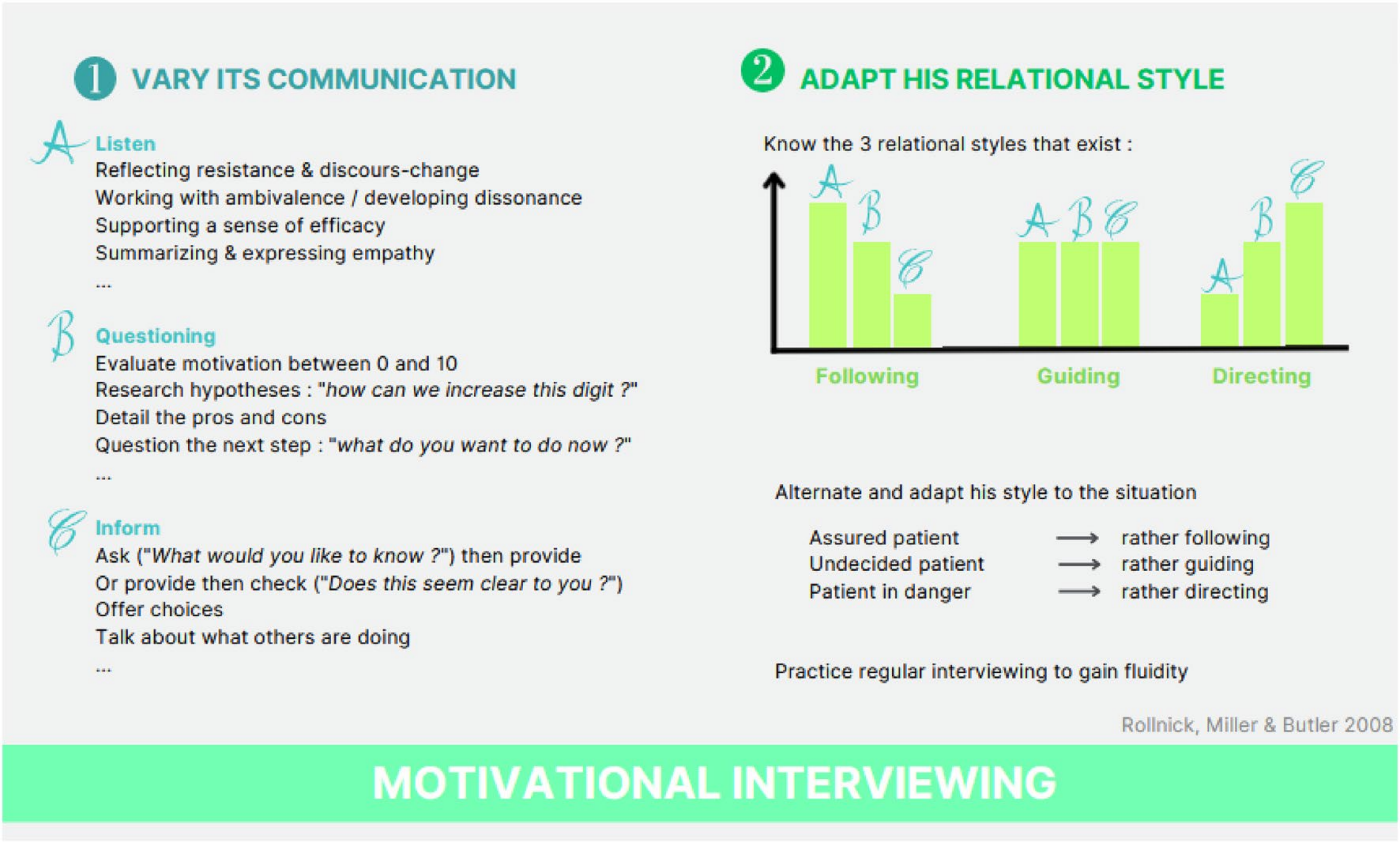
Principles of motivational interviewing: motivational interviewing goal is to increase intrinsic motivation of patient. Text corresponds to useful notions. Graphic might help to understand the different notions described in the text to adapt their relational style

Because there is often a gap between pleasant court-term and unpleasant long-term consequences, motivation is a key factor in care for substance use disorder (DiClemente 1999). Even for patients who agree to engage in care, it is known that only 7–64% will continue at 6 months (Ward et al. 1992). As a professional, one must therefore be attentive to any signs of ambivalence toward treatment and care. The therapy designed to decrease this ambivalence and increase intrinsic motivation for change is called motivational interviewing (Sayegh et al. 2017). In this therapy (Rollnick et al. 2008), therapists are attentive to their communication strategies: following, guiding, and directing styles (Fig. 2).

In theory, motivational interviewing does not necessarily take more time than standard care, according to the designers of the therapy (Rollnick et al. 2008). Thus, motivational interviewing adds no cost to standard follow-up. A meta-analysis referencing all randomized controlled trials on motivational interviewing has revealed that the interventions evaluated ranged from one 15-min session to four 1-h sessions (Burke et al. 2003). In opioid addiction, two models were evaluated. First, Saunders et al. (1995) demonstrated that while only 30% of patients continued care at 6 months after a 1-h educational interview, this rate increased to 49% after a motivational interview of the same duration. Second, Bernstein et al. (2005) showed that a shorter model could also be effective: the opioid abstinence rate was 30.6% at 6 months in the control group against 40.2% in the group that had received a 20-min motivational interview. However, more studies are needed to improve characterization of the efficiency of motivational interviewing in OUD. Indeed, some studies suggest that motivational interviewing is counterproductive with patients who are already motivated. Rohsenow et al. (2004) found that 65% of highly motivated patients who had received motivational interviewing were still consuming opioids 10–12 months later, while this rate was only 50% for highly motivated patients who had not received motivational interviewing. For the least motivated patients, however, motivational interviewing was beneficial. Another study (Jaffray et al. 2014) also explained the lack of statistically significant effectiveness of motivational interviewing by the fact that patients may already have a high level of motivation.

While motivational interviewing does not necessarily require more time than standard care, it does require additional training. Several trainings are possible, with different success rates (Hall et al. 2016). The training that correctly introduced 100% of participants to motivational interviewing contained a 12-h workshop along with 84 h of supervision over 4 years (Forsberg et al. 2010). Another training, thanks to a step-by-step model and videos, significantly reduced the time required (an 8-h workshop and 2–4 h of supervision) but correctly introduced only 81% of participants to the therapy (Martino et al. 2011). In general, a short training is less effective but less expensive. Olmstead et al. (Olmstead et al. 2011) found that prices were approximately $359/person for a 1-h training associated with a sharing of biblio/videographic resources, while the prices were approximately $1648/person for a 15-h training. The success rate was higher in the second case, even though it was only 33%. In all cases, success erodes over time away from training (Miller and Mount 2001).

In summary, studies suggest that motivational interviewing does not replace standard care but complements it when the patient is poorly motivated. Other interventions could also have been considered for this second step (e.g., CBT, contingency management, etc.), but they are not specifically designed to address ambivalence in patients and often require additional training time. In severe addictive processes, motivational interviewing appears to be ineffective, and other tools are thus needed.

### How should we manage in case of severe (poly)addictions?

Of the three studies on motivational interviewing in OUD, none evaluated it in cases of addictive comorbidities except with cocaine use disorder (Bernstein et al. 2005). When addictions become too severe, intrinsic motivation may no longer be enough to stop them. In such cases, it may be interesting to increase extrinsic motivation.

**Fig. 3:**
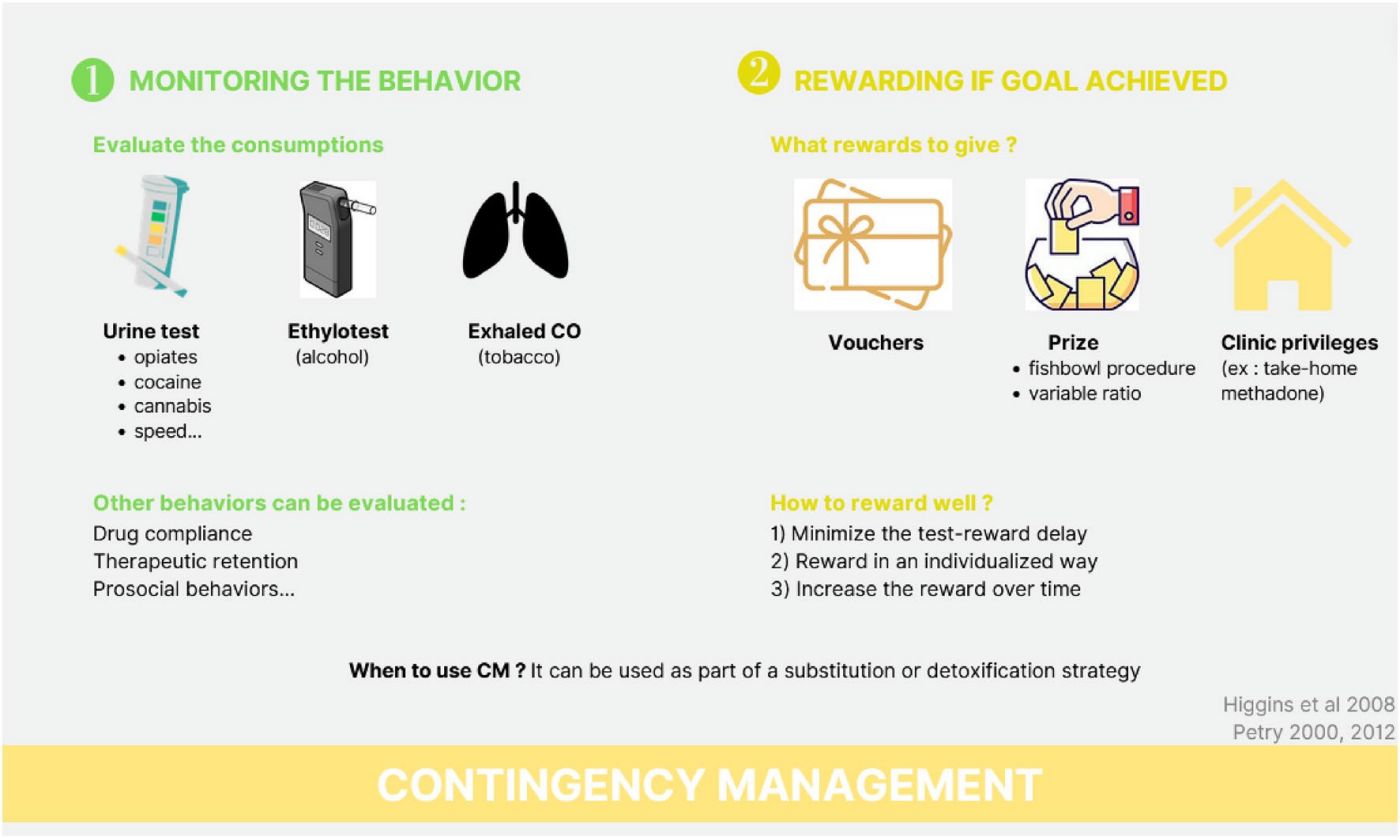
Principles of contingency management: contingency management goal is to increase extrinsic motivation of patient. Text gives the protocol of the therapy. The pictures illustrate the text

The reference psychotherapy for this purpose is contingency management. Contingency management (CM) follows the principle that behavioral changes can be motivated by extrinsic rewards; hence, tangible reinforcers are given to patients if they their drug urinalysis is negative (Stephen et al. 2007; Petry 2000). For this treatment to be effective, three rules must be adhered to (Petry 2013): (1) closely monitor a focal desired patient behavior, (2) provide a tangible positive reinforcer immediately when the behavior occurs, and (3) suspend the reinforcer when the behavior does not occur (Fig. 3). Historically, early methods offered patients convenience and autonomy of take-home medication doses to reinforce drug abstinence (Milby et al. 1978; Stitzer et al. 1977; Stitzeret al. 1980). Contemporary methods derive from studies which use monetary reinforcers in the form of vouchers (Higgins et al. 1993, 1994) or prize draws (Petry et al. 2000).

In the field of addiction, CM has become a reference therapy recommended by many guidelines (Watan Pal et al. 2021), including NICE (2007). In Rice’s meta-analysis (Rice et al. 2020), CM was not only one of the most studied psychotherapies in OUD, with five studies, but also the only one showing statistically significant improvement in therapeutic retention. Its odds ratio (OR = 2.01) outperformed that of motivational interviewing (OR = 1.71). Including all randomized controlled trials of CM in patients treated with OAT, Bolivar et al. (2021) even referenced 74 studies in their meta-analysis. The effect size was in favor of CM for reducing both opioid use (*d* = 0.58) and polysubstance use (*d* = 0.46) and reached 0.75 for therapeutic adherence and 0.70 for psychostimulant use.

Despite these studies, several barriers exist. As with OAT, many people think that the underlying cause of addiction remains unchanged and, therefore, that CM is ineffective or would even decrease a user’s intrinsic motivation to abstain (Cameron and Ritter 2007; Henggeler et al. 2008; Kirby et al. 2006; Rash et al. 2012). Even if a greater receptivity is present in the services that deliver OAT (Hartzler et al. 2012, 2014), only 12% of accredited addiction clinics successfully implement CM in the long term (Roman et al. 2010). To facilitate the implementation, a 1.5-day training is interesting. According to a study (Henggeler et al. 2013), this training improves knowledge of CM and modifies misperceptions among professionals, especially those who are key to the implementation of CM. Participants are satisfied and identify training as a determining factor for the implementation of CM.

Follow-up usually consists of urine testing approximately twice a week and then discussion of the result for 3–5 min with a professional. On average, this follow-up lasts 13.9 weeks, and the maximum reward is $10.25 in OUD studies, according to the meta-analysis by Bolivar et al. (2021). The Washington State Institute (Washington State Institute for Public Policy 2019), which considers several economic studies such as one by Peirce et al. (2006), estimates the price of this therapy between $374 and $601. According to the same institute, this price is generally profitable in view of its effectiveness in 59–77% of cases, with savings amounting to $3895 and $23,016. A more recent systematic review even found that for some 3-month programs, the probability of being cost-effective could reach 88.4% (Shearer et al. 2023). CM prize formats increase this rate of return by reducing the cost of rewards (e.g., $203 for 3 months; Petry et al. 2005).

Regarding the literature on adolescents, Lott and Jencius (2009) demonstrate the efficacy and cost-effectiveness of CMAs for adolescents who are very sensitive to immediate reward; this approach is ideal for them. To introduce evidence-based practices for the treatment of SUDs in adolescents, Henggeler et al. (2007, 2008) led studies on training for CM. The results suggested that organizations and clinicians were interested in CM, and this interest translated into implementation efforts on the part of the clinicians. Indeed, 58% of eligible clinicians reported implementing CM with at least one patient following the training.

The limitation of contingency management is that few studies exist on comorbidity related to SUD and psychiatric disorders, and, to our knowledge, none exist on contingency management in patients with psychiatric disorders without addictions. Contingency management involves easily monitoring problematic behavior, but this is more difficult outside of addictions. Existing studies seem to show that contingency management acts partially on drug-related psychiatric symptoms, such as depression and anxiety (Petry et al. 2013), and remain cost-effective in cases of comorbidities with a psychiatric disorder (Murphy et al. 2015).

In summary, contingency management benefits from many effectiveness studies in opioid use disorder. Incorporating extrinsic motivation can be an additional aid for patients without sufficient intrinsic motivation. As CM requires more time and professionals specifically trained in this intervention, the therapy should be reserved for more severe addictions. However, to confirm the benefits of CM, studies on psychiatric comorbidities are lacking.

### How should we manage in case of a dual diagnosis?

Treatment for addiction partially reduces psychiatric comorbidities (Kertesz et al. 2006). However, if there are severe psychiatric comorbidities associated with OUD (26.9% of OUD cases, according to Jones and McCance-Katz (2019), targeting motivation may be insufficient, and implementing a contingency management protocol may be challenging. One study showed that the effectiveness of addiction contingency management decreased with the severity of psychiatric comorbidity (Weinstocket al. 2007) unless standard treatment was added.

In psychiatric disorders, standard treatment generally involves cognitive-behavioral therapy (CBT), as recommended by guidelines (NICE 2011; Reddy et al. 2020). These recommendations are also relevant for OUD (Watan Pal et al. 2021). Many meta-analyses exist on CBT for both psychiatric disorders (López-López et al. 2019) and SUD/OUD (Ray et al. 2020; Wen et al. 2023). Learning strategies to remain sober appear to be the main mechanism of action in the SUDs (Magill et al. 2020). Compared with CM, which acts quickly and intensely, CBT acts more slowly but more durably (Rawson et al. 2002). In case of a dual diagnosis, CBT acts on both addictions and comorbid psychiatric disorders (Iqbal et al. 2019; Lees et al. 2021; Roberts et al. 2022). Psychiatric disorders are also additionally managed through adapted medications.

**Fig. 4:**
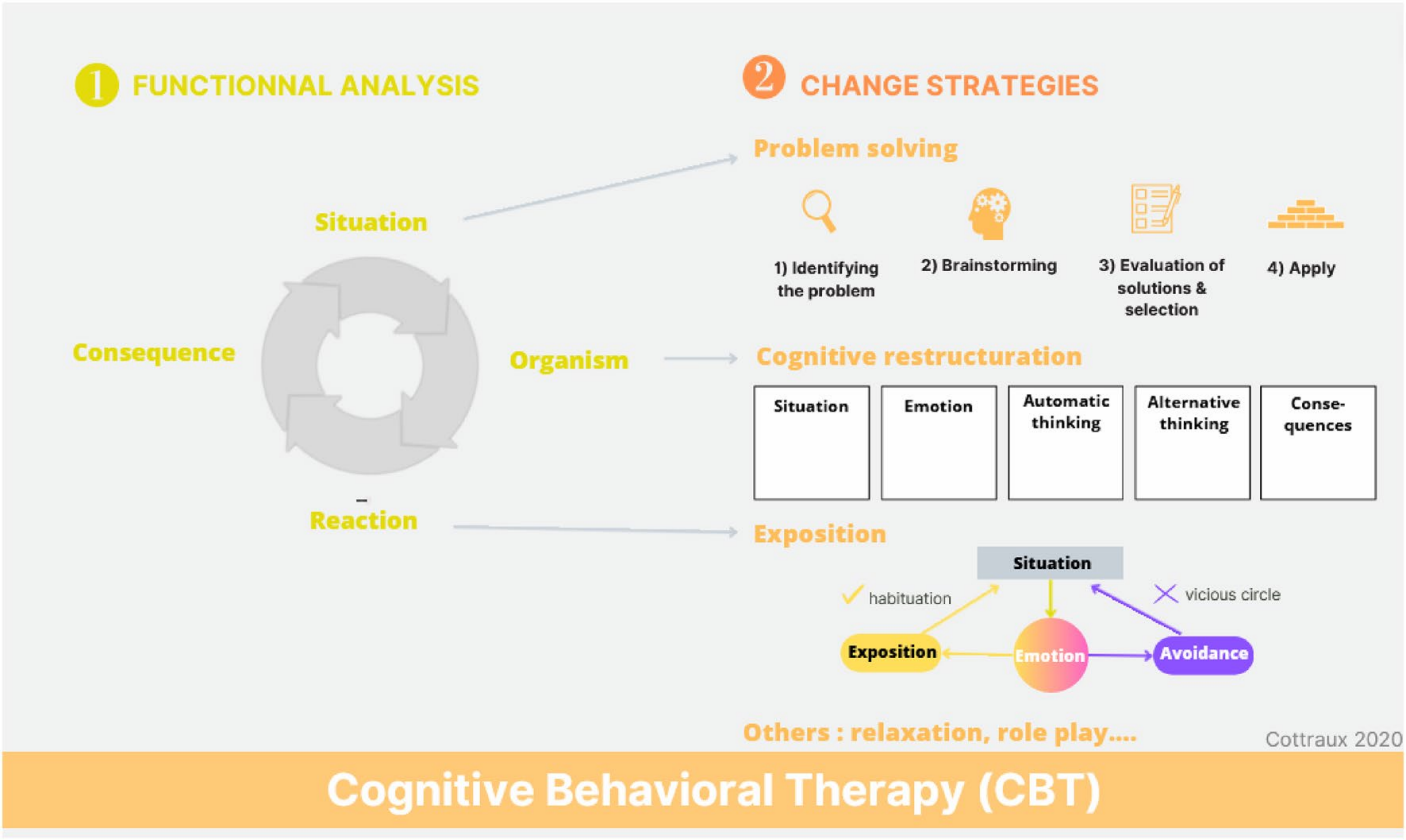
Principles of cognitive-behavioral therapy: cognitive-behavioral therapy goal is to learn about patient’s conditioning process and the change strategies. Text shows analysis and strategies. The schemas describe useful tools with patient. Logos help memorize

This simultaneous effectiveness of CBT and CM is explained by the principle of the therapy. Although CM modifies the conditionings from the outside, CBT helps the patient to identify and modify their own conditioning (Cottraux 2020). Functional analysis highlights vicious circles reinforcing dysfunctional behaviors, whether they are related to opioid use disorders or psychiatric comorbidity. These vicious circles involve emotions, thoughts, and behaviors. By detailing them, functional analysis thus enables one to identify therapeutic strategies (Fig. 4). Several tools are used to this end: cognitive restructuring exposure, problem solving, relaxation, and role play.

Unlike previous interventions, CBT requires long training. Specific master’s degrees in CBT exist and provide a solid foundation for treating different psychiatric disorders (Henrich et al. 2023). As this intervention requires time and practical supervision, the cost is $1485 per participant for an in-person workshop and supervision or $785 for a distance workshop and supervision, and it can even decrease to $440 for online training or $145 for training via a manual along with an orientation session (Valenstein-Mah et al. 2020).

In addition to the cost of training, CBT is also expensive to practice. According to a recent meta-analysis on addictions, CBT lasts on average 16 sessions (Ray et al. 2020), although it can be longer or shorter depending on the clinical severity and the presence of comorbidities. This meta-analysis found a range of four to 48 sessions, with a CBT session generally lasting between 30 and 60 min. Given the knowledge required, the sessions are mainly performed by psychiatrists or psychologists; however, in some cases, they may also be performed by other physicians or nurses who are trained to treat addictive disorders (Compton and Blacher 2020). CBT in OUD is estimated on average at $567 and is generally cost-effective in 49% of cases (Washington State Institute for Public Policy 2019). For other substance use disorders, this cost decreases to $279 and is cost-effective in 56% of cases. Computer protocols also exist and enable a reduction in costs, even if the effectiveness seems lower (López-López et al. 2019).

The difficulty with CBT is that it refers either to several scientifically validated psychotherapies (e.g., motivational interviewing, contingency management, exposition, etc.) or to a psychotherapeutic protocol (which involves performing a functional analysis and then using change strategies). We must therefore be careful because some reviews use the term CBT in the first sense, not in the second, such as McHugh et al. (2010), who integrated both relapse prevention and contingency management. Recent reviews, however, use the second definition instead (Rice et al. 2020). When examining CBT studies in the second direction, the downside is that they tend to exclude suicidal patients (Brooks et al. 2021).

In summary, CBT is adapted to treat both OUD and its psychiatric or addictological comorbidities. However, CBT studies tend to exclude patients with high suicidality.

### How should we do in case of recurring self-harm behaviors?

As mentioned in the introduction, 15–21% of overdoses may correspond to a form of suicidal behavior (Oquendo and Volkow 2018; Johnson et al. 2013).

**Fig. 5:**
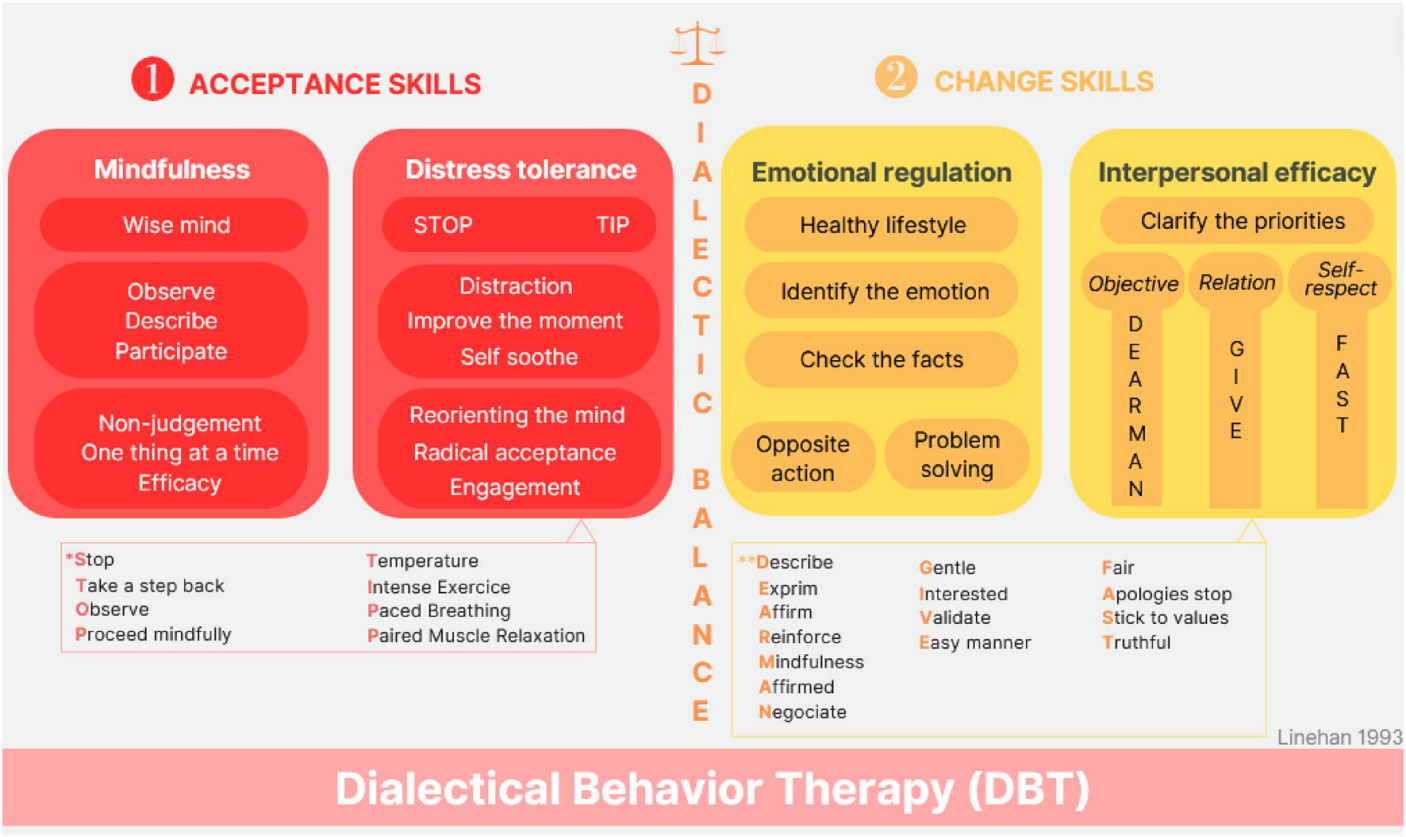
Principles of dialectical-behavior therapy: dialectical-behavior therapy goal is to develop with patients’ acceptance and change skills. Text describes skills categorized in 4 modules (mindfulness, distress tolerance, emotional regulation, interpersonal efficacy)

The first therapy to show positive results in borderline personality disorder (BPD) (Linehan et al. 1991), dialectical-behavior therapy (DBT), is now a reference therapy for this disorder and more broadly for treating repeated suicidal behaviors (Storebø et al. 2020). In the 1980s, Marsha Linehan noticed that CBT taught at the time was not effective for chronic suicidal patients because it was too change-oriented. With DBT, Masha Linehan proposed balancing change skills and acceptance skills in a dialectic manner (Linehan 1993a, b). This dialectic balance helps patients to decrease impulsive behavior by employing several acceptance-oriented skills (e.g., mindfulness, distress tolerance) and to improve decision-making by employing several change-oriented skills (e.g., emotional regulation, interpersonal effectiveness) (Fig. 5). To enhance patients’ capabilities, skills training is key in DBT programs. A study confirmed the central role of the learning of skills in the therapy (Linehan et al. 2015), and patients who learn and practice these skills the most improve the most in terms of suicidal behaviors (Neacsiu et al. 2010). As in adults, DBT has also been shown to be effective for adolescents with borderline personality disorder, emotion regulation disorders, and/or suicidal behaviors (Rodante et al. 2023).

Regarding substance use disorders, there are many studies on DBT to treat various addictions and psychiatric comorbidities with chronic suicidality (Giannelli et al. 2019; Warner and Murphy 2022). These studies generally show an improvement in suicidal behavior after DBT, especially in two studies with 10% opioid addiction Linehan et al. 1999) and 22% opioid addiction (van den Bosch et al. 2002, 2005; Verheul et al. 2003), respectively. In these two studies, the therapeutic disruption rates were 36% and 37%, respectively, in the DBT groups, while they were 73% and 77%, respectively, in the control groups. Several techniques exist to promote therapeutic adherence in DBT (Bornovalova and Daughters 2007).

For OUD specifically, two randomized controlled studies exist. The rate of treatment dropout was 18% in the first study (Linehan et al. 2002) and 8% in the second (Rezaie et al. 2021). Authors show an improvement in substance use, emotional regulation, and distress tolerance. Although these criteria are mainly related to suicidal behavior (Gratz and Roemer 2004), direct assessment of their evolution is lacking. In addition, the studies did not distinguish overdoses from other suicidal modalities. Only one case report specifies the evolution of overdoses in patients with borderline comorbidity (Dimeff et al. 2000). More robust studies are therefore needed, even if some authors suggest that the same therapeutic strategies could be applied for suicide attempts as for overdoses (Farrell et al. 1996).

Standard DBT is relatively intensive over 1 year and includes four therapeutic modalities: a 2-h-per-week skills training group, a 1-h-per-week individual therapy, a team consultation, and telephone coaching. Global societal costs decreased from $30,463 the year before DBT to $20,389 in the year of DBT, and then to $1603 the year after DBT (Wagner et al. 2014). The cost of DBT itself is $6013. This cost is mainly related to individual therapy ($3659), followed by team consultations ($1167), skills training groups ($1104), and finally telephone coaching ($81). To reduce costs, DBT can be practiced in a 4-month standalone group format for 2.5 h per week (Delaquis et al. 2022; Valentine et al. 2015). The DBT for Adolescents (DBT-A) program (Rathus and Miller 2014) features condensed (12 weeks of therapy) and simplified sessions (Rathus and Miller 2014). The content includes the four competency modules developed for adult DBT, along with a fifth module targeting relationships with the family and environment. In addition, it is possible to hold these DBT group sessions online to reduce practical constraints (Lakeman et al. 2022) and/or transdiagnostically to reduce recruitment difficulties (Durpoix et al. 2023).

The reference training lasts 2 × 5 days over 6 months (DuBose et al. 2018; Landes and Linehan 2012), is offered notably by the Association for Psychological Therapies, and costs $1755 per person (DuBose et al. 2018). In practice, only 33% of DBT therapists manage to follow such a long training (Landes et al. 2016); the majority have followed a training of 1–2 days (74%), and almost all have read the reference manual (97%). To fully understand the dialectical principles formalized in DBT, one should ideally already have mastered the basic principles of CBT. The problem is that the number of professionals trained in DBT is small compared with clinical needs (Iliakis et al. 2019). Solutions exist such as integrating trainings during medical studies to sensitize future professionals to the use of DBT strategies (Frederick and Comtois 2006).

In summary, DBT benefits from numerous studies of chronic suicidal patients and patients suffering from addictive disorders. Incorporating dialectical balance helps these patients to stabilize. Given its high cost, standard DBT becomes cost-effective when patients frequently require emergency care services. In OUDs, the therapy is therefore best reserved for patients with chronic suicidality. However, it could be opened to other complex dual diagnoses for which the benefit might be examined, such as PTSD associated with addictive disorders.

## Discussion

In our review, we highlighted that OAT only, counseling, motivational interviewing, contingency management, and cognitive-behavioral therapy are the most studied therapies although a recent systematic review on psychosocial interventions adjunctive to opioid agonist therapy (Rice et al. 2020) has listed 37 psychosocial interventions. Our work shows that psychotherapies might be adapted as a function of the complexity of OUD. Our results suggest that counseling is an efficacious psychosocial intervention for opioid users motivated to receive opioid agonist treatment and without poly-addictions and psychiatric comorbidities. It involves a medical evaluation and follow-up to assess whether a patient is motivated by OAT, to provide information on the correct intake of the medication, and to ensure that the patient follows the recommendations. For other conditions, we argue that specific psychotherapies might be required. In case of ambivalence regarding care and treatment for OUD, the literature indicates that motivational interviewing might be a tool to enhance patients’ motivation by weighing up the benefits and risks of consumption and identifying factors conducive to reducing/stopping consumption. In case of severe addiction, CM might help increase patients’ chances of stopping opioid use by rewarding users for their abstinence. Indeed, according to our results, CM has become a reference therapy recommended by many guidelines (Watan Pal et al. 2021), including NICE (2007), with a significant improvement in therapeutic retention and good efficiency for severe addiction and poly-addiction. In case of psychiatric comorbidities, we found that CBT might be efficient for both psychiatric comorbidities and opioid use. The goal of this therapy is to analyze dysfunctional behaviors and move toward more functional behaviors. Dysfunctional behaviors are key symptoms in a dual diagnosis because of the implementation of drug use to alleviate psychiatric symptoms. However, in case of suicidal behaviors, CBT would be a limited form of treatment’. In this case, our results indicate that DBT improves patient retention in care and decreases suicidality and emotional dysregulation, which contribute to addictive disorders. Our results highlight that the complexity of opioid use disorder requires tailored treatment by a trained/formed professional and structured care. These factors, however, increase costs.

**Fig. 6:**
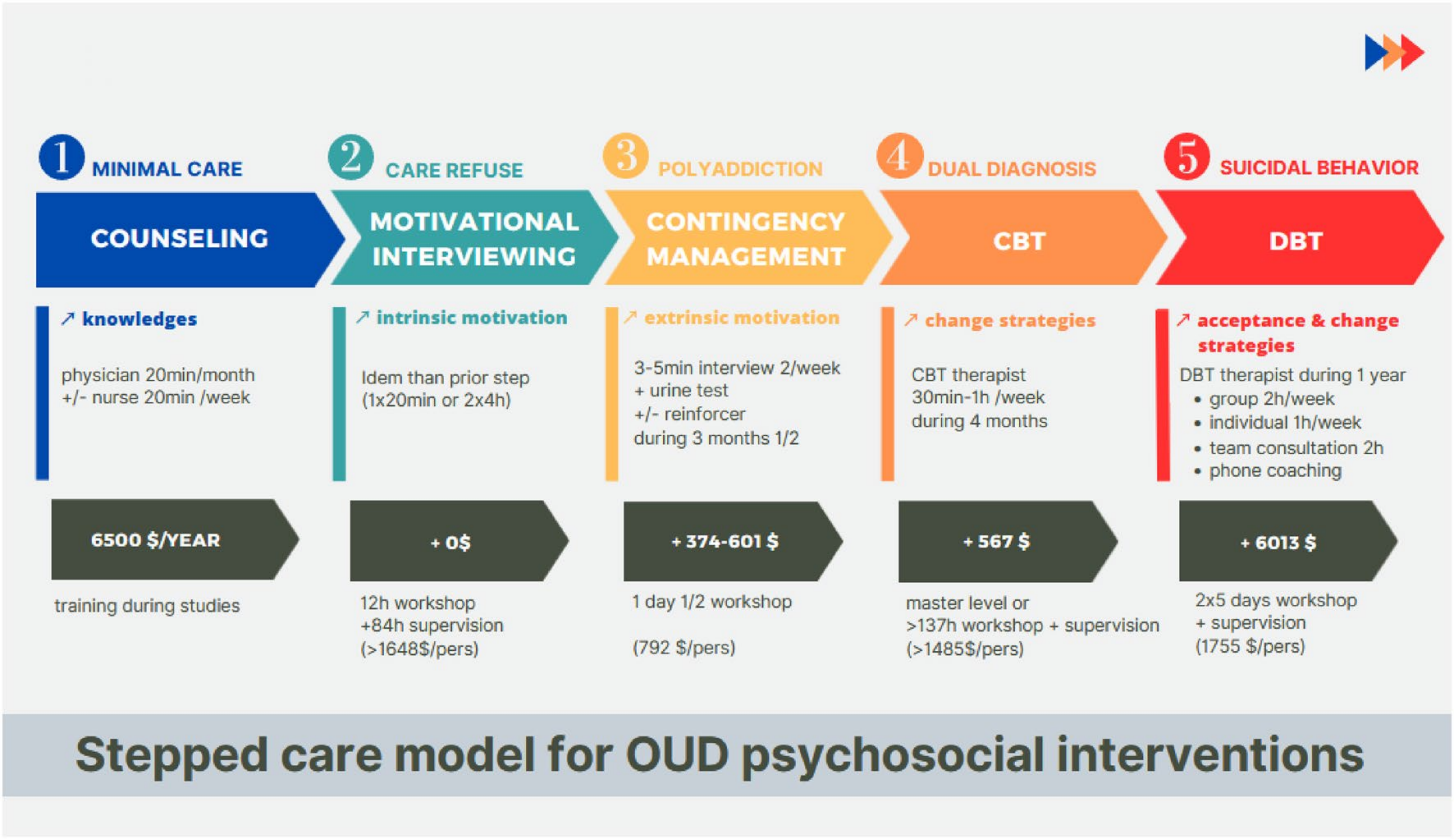
Stepped-care model for OUD psychosocial interventions: stepped-care goal is to research the best care depending on severity. On the figure from top to bottom: line 1 corresponds to clinical severities; line 2 corresponds to proposed psychotherapies; line 3 corresponds to psychotherapy goals; line 4 corresponds to practice time; line 5 corresponds to psychotherapy costs; line 6 corresponds to training time (+ cost of training)

These results led us to propose a stepped-care model to aid clinicians in finding the best psychotherapeutic strategy depending on the complexity of the patient’s diagnosis. For a pharmaco-therapeutic strategy, the guidelines already recommend a specific stepped-care model (buprenorphine-naloxone in first line and methadone in second line), as its effectiveness has been demonstrated in a randomized controlled trial (Kakko et al. 2007). The stepped-care model aims to reduce the time required for a specialist therapist without compromising effectiveness (Bower and Gilbody 2005). This is a system of delivering and monitoring treatments, such that the most effective, least intrusive, and least resource-intensive treatments are delivered first. Patients can be ‘stepped up’ if initial treatments become insufficient or ‘stepped down’ if initial treatments become excessive. This approach has been increasingly promoted for the management of mental disorders (NICE 2022). However, for a psychosocial strategy in OUD, the guidelines provide no details except that an intervention must be proposed. The only available study found that psychosocial strategy was inferior to performing behavioral psychotherapy (Brooner et al. 2007). The disadvantage of the model evaluated in this study is that it proposes more quantitative than qualitative change, whereas models validated in other disorders generally use a greater diversity of psychosocial interventions. Here, we propose constructing this model by solving three problems, which complicate the treatment of patients with OUD. First, it is known that patients with addiction, particularly opioid addiction, frequently experience difficulties remaining in long-term care and need a motivational approach. Second, addiction or psychiatric comorbidities are frequent and increase the risk of overdoses (Ranapurwala et al. 2018; Webster 2017). Third, some patients regularly put their lives in danger with repeated overdoses or suicide attempts (Johnson et al. 2013; Oquendo and Volkow 2018). This work allows us to propose the following stepped-care model of psychotherapies for OUD (see Fig. 6), with five key questions guiding our reasoning. Each psychosocial intervention used in this model has at least two randomized controlled trials regarding opioid use disorder, providing an interesting empirical basis for reflection. In addition, consideration of the principle of therapies helps answer the five key questions. Counseling is sufficient for patients who are already motivated (step 1), motivational interviewing for patients who lack intrinsic motivation (step 2), contingency management for patients who also require extrinsic motivation (step 3), CBT for patients who have psychiatric problems aside from motivation (step 4), and DBT for patients with suicidal urges (step 5).

Our model is consistent with stepped-care models for other psychiatric disorders. It proposes a gradation of care according to patient severity. Each new step in our model requires more intensive follow-up time and training, in order to treat more severe patients. It shares certain similarities to the stepped-care model for depression (NICE 2022; Rivero-Santana et al. 2021). In both cases, the first step is based on basic principles (counseling for OUD and psychoeducation for depression), while one of the intermediate steps involves a behavioral intervention (contingency management for OUD and behavioral activation for depression) or CBT, and the final step is a high-intensity psychological intervention. Our model is thus more comprehensive than the previous stepped-care model of psychosocial interventions evaluated in opiate addiction (Brooner et al. 2007). Its originality lies in the fact that it seeks to use the various psychosocial interventions available in the literature to better adapt to each clinical profile. This model is also interesting for adolescent care because it could be adapted with a modular approach which allows addictology teams to add specific components to care for adolescents. We propose that adaptations of the stepped-care model for adolescents include three components: (1) a module for parents (psychoeducation, parental guidance), (2) a family therapy module, and (3) an adolescent ‘community reinforcement’ module (Godley et al. 2014). From a care organization point of view, each team can organize itself as it wishes, for example by integrating child psychiatry caregivers for the three adolescent-specific components. Moreover, the treatment of adolescents is not optimal, and despite recommendations, drug treatments are less well prescribed in adolescents than in adults. For example, only 18% of adolescents admitted for treatment due to heroin use in 2015 in the USA had a treatment plan that included treatment with buprenorphine/naloxone or methadone, compared with 42% of adults (Mental Health Report). This model thus offers new possibilities for interventions that can be implemented by optimizing the resources of addictology in collaboration with child psychiatry departments.

However, given the difficulty in training and mastering these different interventions, these five steps (see Fig. 6) cannot be practiced by all professionals. Logically, the higher the level of specialization of the place of care, the higher the level of intervention should be proposed. Counseling should be mastered by all professionals who are likely to treat opiate addictions; motivational interviewing, at least by professionals specializing in addictions; contingency management and CBT, at least by specialized centers; and DBT, at least by university centers. For patients to receive effective care, professionals must be trained, as they are more optimistic concerning treatment of substance use disorder if they have training and personal/professional experience (May et al 2002). The difficulty is that few physicians engage in SUD training upon completion of their studies (Sharfstein and Olsen 2019). An important reason is insufficient training of healthcare professionals (Madras et al. 2020). Several negative attitudes toward patients with SUD exist among physicians, nurses, psychologists, social workers, and other health professionals. SUD is often seen as a choice (Wakeman et al. 2016); OAT as ‘replacing one addiction with another’ (Yasgur 2020); and patients with SUD as violent, manipulative, and unmotivated (van Boekel et al. 2013). Many clinicians cite these attitudes as a major reason for not providing care to this patient population (Haffajee et al. 2018; Wakeman et al. 2016). Professionals with these attitudes are less engaged and empathetic with these patients (van Boekel et al. 2013). Moreover, these negative attitudes among providers decrease patients’ engagement in treatment (Earnshaw et al. 2013; van Boekel et al. 2013). A solution would be to deliver better education about SUD and treatment experience in graduate-level studies. In a study, compared with psychiatrists who had not received buprenorphine training during residency, those who had were more confident treating opioid addiction (84.6% vs 46.3%), more likely to view opioid addiction as a treatable illness (98.1% vs 75.6%), and more able to prescribe buprenorphine (Suzuki et al. 2014). Another solution is to improve organizational and role support structures for health professionals. To implement a stepped-care model as a strategy of care for OUD, professionals must be trained in these different psychotherapies.

## Limitations

In our work, we propose graduating psychotherapies according to the level of complexity of OUD. Doing this would require bringing in the same unit psychotherapists trained in many different psychotherapies. Moreover, other psychotherapies, such as those inspired by psychoanalysis, are not well evaluated. However, this does not mean that they are without interest; rather, their place remains to be determined to enrich the stepped-care model.

## Conclusion

OUD is a dramatic public health problem, and its treatment requires opioid agonist treatment associated with psychotherapies. We examined the role of the most efficient psychotherapies according to severity and complexity of OUD (i.e., psychiatric comorbidities and suicidal behaviors). We proposed a stepped-care model for treatment of both adults and adolescents but with adaptations for the latter. For complex dual disorders, professionals must be trained and psychotherapies must be structured, both of which bear costs. In this sense, collaboration among specialized structures with trained professionals and different approaches is fundamental to offer an adapted treatment according to the complexity and severity of OUD.

## Data Availability

Data availability does not apply to this article. For further information, please contact the authors.
